# Grain Boundary Interfaces Controlled by Reduced Graphene Oxide in Nonstoichiometric SrTiO_3-*δ*_ Thermoelectrics

**DOI:** 10.1038/s41598-019-45162-7

**Published:** 2019-06-13

**Authors:** Jamil Ur Rahman, Nguyen Van Du, Woo Hyun Nam, Weon Ho Shin, Kyu Hyoung Lee, Won-Seon Seo, Myong Ho Kim, Soonil Lee

**Affiliations:** 1 Energy & Environmental Materials Division, Korea Institute of Ceramic Engineering & Technology, Jinju, 52861 South Korea; 2 School of Materials Science and Engineering, Changwon National University, Changwon, 51140 South Korea; 3 Department of Materials Science and Engineering, Yonsei University, Seoul, 03722 South Korea

**Keywords:** Thermoelectric devices and materials, Thermoelectrics

## Abstract

Point defect or doping in Strontium titanium oxide (STO) largely determines the thermoelectric (TE) properties. So far, insufficient knowledge exists on the impact of double Schottky barrier on the TE performance. Herein, we report a drastic effect of double Schottky barrier on the TE performance in undoped STO. It demonstrates that incorporation of Reduced Graphene Oxide (RGO) into undoped STO weakens the double Schottky barrier and thereby results in a simultaneous increase in both carrier concentration and mobility of undoped STO. The enhanced mobility exhibits single crystal-like behavior. This increase in the carrier concentration and mobility boosts the electrical conductivity and power factor of undoped STO, which is attributed to the reduction of the double Schottky barrier height and/or the band alignment of STO and RGO that allow the charge transfer through the interface at grain boundaries. Furthermore, this STO/RGO interface also enhances the phonon scattering, which results in low thermal conductivity. This strategy significantly increases the ratio of σ/κ, resulting in an enhancement in ZT as compared with pure undoped STO. This study opens a new window to optimize the TE properties of many candidate materials.

## Introduction

Thermoelectric (TE) devices possess the capability to capture heat from the waste heat and convert it into useful electricity, which benefits to the improvement of energy efficiency, and reduces environmental pollution. The performance of a thermoelectric device is determined by the thermoelectric dimensionless figure-of-merit, ZT = S^2^σT/κ, where S, σ, T and κ are the Seebeck coefficient, electrical conductivity, the temperature in Kelvin, and thermal conductivity, respectively^1,2^. To date many states of the art TE materials like, Bi_2_Se_3_, Bi_2_Te_3_, PbTe, and Skutterudites based materials have been developed with highest ZT and successfully used for TE conversions. However, the high-cost, stability and toxicity stuck them for commercial applications^3–6^. On the other hand, it is believed that oxide-based TE materials have been considered to be the best counterpart due to their low cost, less ecological concerns, and potential for high-temperature stability but the TE performance of these materials are far from the practical applications^7,8^. Therefore, it is essential to find new oxide thermoelectric materials with a high ZT value.

**Figure 1:**
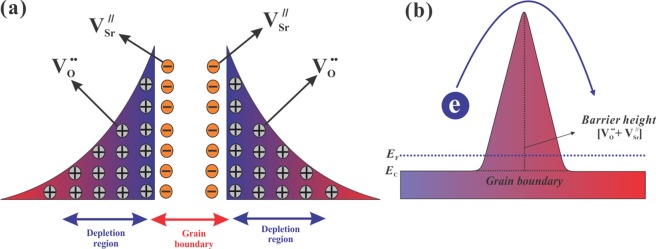
Schematic of (**a**) double Schottky barrier and (**b**) double Schottky barrier height at a grain boundary.

In recent years, strontium titanium oxide (STO) is particularly considered an interesting *n*-type material in thermoelectric research due to its high absolute Seebeck coefficient^9^. Thus, many efforts have been made to enhance its electrical conductivity, such as cationic and anionic substitution, but ZT is still not competitive because of the high lattice thermal conductivity (*κ*_L_)^10–14^. Nano-structuring, doping, and creating oxygen vacancies in STO provide an opportunity to decrease the lattice thermal conductivity through phonon scattering without noticeably affecting their electrical conductivity^15–19^. These strategies were mainly focused on increasing electrical conductivity and decreasing the lattice thermal conductivity, but still, the thermoelectric performance of STO for practical application remains a challenging task. Unfortunately, the possibility to control the formation of the double Schottky barriers and its associated depletion regions at the grain boundaries were mostly missed by the researchers, which had been one of the victims of such impediment in the TE performance of STO^20,21^. In this double Schottky barrier, electrons are trapped at the grain boundaries which cannot contribute to the electrical conduction and act as a scattering center for electrons mobility. In polycrystalline STO, those double Schottky barriers are formed due to the space charges produced by strontium vacancies (${V}_{Sr}^{^{\prime\prime} }$) and oxygen vacancies (${V}_{O}^{\cdot \cdot }$) at the grain boundaries, as shown in the schematic Fig. 1(a)^21–24^. The double Schottky barrier is highly resistive, and the mobility of the whole sample can be hindered by the presence of the barrier as shown in the schematic Fig. 1(b). So, it is important to control the double Schottky barrier and its associated depletion region to boost the performance of such thermoelectric materials. To address this challenge, an effective approach is required to identify the suitable components. The possible solution could be the hybrid interface control through the incorporation of highly conductive materials at the grain boundaries which would lead to deterioration of the double Schottky barrier and its associated depletion region^25^. The deterioration of the double Schottky barrier would change the electronic transport properties at atomic-scale^26^.

To demonstrate this issue, herein we propose a novel strategy to achieve enhanced transport properties with the improved thermoelectric performance of undoped STO by fabricating its composite with reduced graphene oxide (RGO). Graphene has received substantial attention since its discovery by Novoselov *et al*.^27^ due to its unique electrical conductivity, high specific surface area, and excellent mechanical properties^28–31^. Moreover, Graphene has zero band gap, easy to be dispersed and its composite with other semiconducting materials results in inter-material charge transfer. In this regard, there are some recent trails of the fabrication of STO-graphene composites for various applications^32–34^. Those studies suggest that graphene would be a prominent candidate material to mix with undoped STO matrix. In addition to this, this approach can prepare large amounts of bulk samples without using any hazardous gases such as H_2_ and CO in the annealing process^35^.

Refer to the above scenario, to date, no work is available to control the double Schottky barrier of polycrystalline STO. Herein we demonstrate a promising preparation approach to fabricate STO/RGO composite free from the double Schottky barrier. Most importantly, by this strategy even in the undoped polycrystalline STO can provide single crystal-like charge transport properties with high electrical conductivity and power factor. Moreover, this approach also provides the opportunity to suppress the phonon transport at the STO/RGO interface.

## Results and Discussion

**Figure 2:**
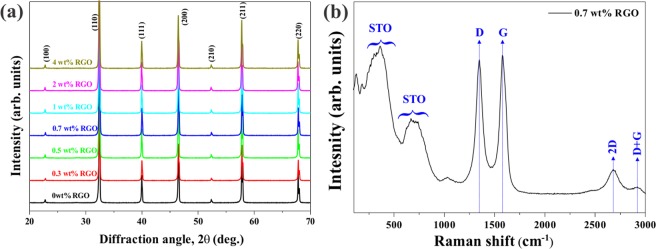
Room temperature (**a**) XRD patterns of SrTiO_3_-RGO (0–4 wt% RGO) composites and (**b**) Raman spectra of STO-0.7 wt% RGO composite.

Figure 2(a) represents the X-ray diffraction (XRD) patterns of STO-RGO composites. The observed patterns suggest the formation of a single-phase perovskite structure and all diffraction peaks were indexed to the cubic phase of STO. The peak positions of STO were not influenced by the RGO content, and without any trace of secondary phases in the XRD patterns. Furthermore, the RGO peaks could not be observed in the XRD pattern, which is due to the low concentration of RGO contents. To confirm the presence of RGO in STO-RGO composites, Raman spectra was used, as shown in Fig. 2(b). The Raman spectrum at 1350 cm^−1^ (D-band centered) and at 1597 cm^−1^ (G-band centered) indicates the existence of RGO and suggests that the SPS process has not damaged RGO. In addition to this, the Raman spectrum at 200–400 cm^−1^ and 600–750 cm^−1^ is related to the second order of Raman active mode of STO^36^.

**Figure 3:**
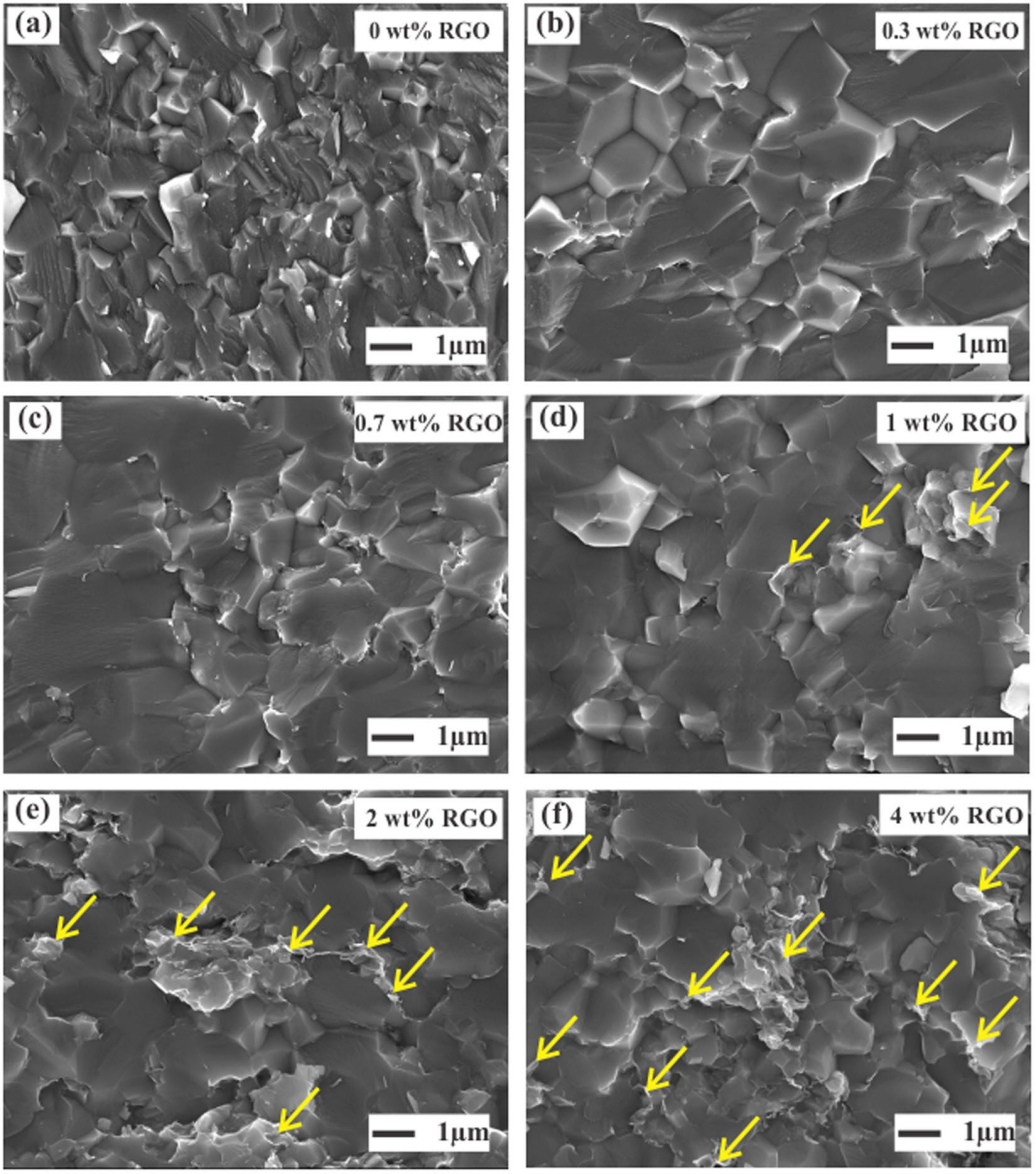
Microstructural characterization of the fractured SrTiO_3_-RGO (0–4 wt% RGO) composites. Yellow arrows in (**d–f**) represent the agglomerated RGOs.

**Figure 4:**
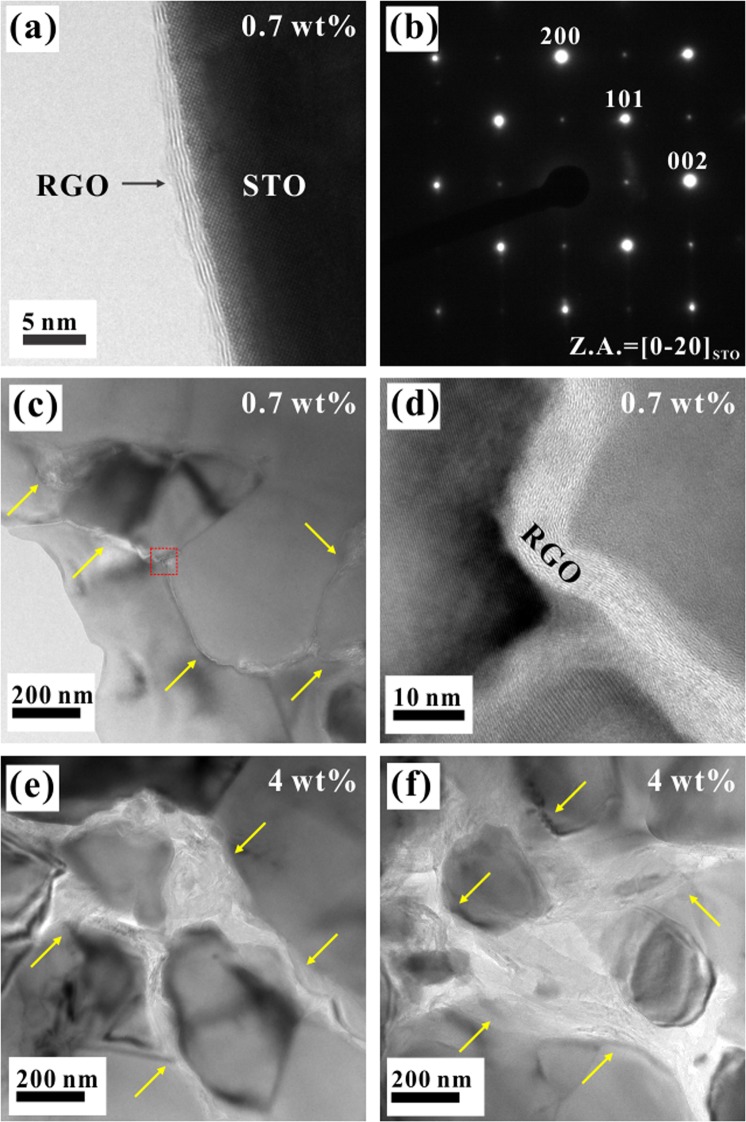
Microstructural characterization of the SrTiO_3_-RGO composites; (**a**) HRTEM micrograph of the SrTiO_3_-RGO hybrid powder, (**b**) diffraction pattern, (**c,d**) low and high magnification micrograph of the SrTiO_3_–0.7 wt% RGO composite, and (**e,f**) low and high magnification micrograph of the SrTiO_3_-4 wt% RGO composite. Yellow arrows represent the RGO at the grain boundaries.

The morphological features of the fractured STO-RGO composites and the distribution of RGO in the STO matrix were analyzed by the scanning electron microscopy (SEM), as shown in Fig. 3(a–f). A similar microstructure for all RGO contents can be anticipated, having similar average grain size and relative densities of (>95%). Furthermore, no agglomeration of RGO ≤ 0.7 wt% could be observed in the SEM micrographs, indicating that undoped STO grains well interacted with RGO sheet. However, for higher RGO contents ≥1 wt% some of the RGO are agglomerated at the grain boundaries as indicated by the arrows in Fig. 3(e,f). To further confirm the presence and distribution of RGO at the interface of undoped STO matrix, transmission electron microscopy (TEM) was used as shown in Fig. 4(a–f). Figure 4(a) confirms that RGO has well coated on the surface of undoped STO particles. The electron diffraction pattern of the selected area is presented in Fig. 4(b). The observed pattern is well-matched with a cubic STO crystal. Furthermore, on the one hand, it can be seen in Fig. 3(c) that STO/RGO (0.7 wt%RGO) grain boundaries have well covered by thin RGO layers and this interfacial structure of the composites can be more clearly seen in HRTEM micrograph Fig. 4(d). On the other hand, for high RGO content (4 wt%), the RGOs are agglomerated at the grain boundaries as indicated by the arrows in Fig. 4(e,f), which is consistent with the SEM investigations.

**Figure 5:**
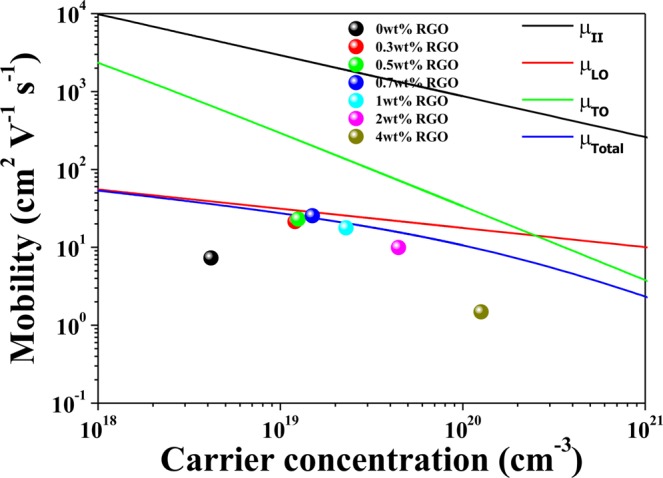
Room temperature calculated mobilities for reduced single crystal STO as a function of carrier concentration and its comparison with STO-RGO composites.

Figure 5 represents the room-temperature Hall mobilities of the STO-RGO composites and its comparison with calculated single crystalline mobility vs. carrier concentration, shown by solid lines. It is interesting to see that the mobility of STO was significantly enhanced by the incorporation of optimum RGO concentration and is similar to single crystalline STO. As discussed above, it is believed that  this significant enhancement in the mobility is due to the weakening of double Schottky barrier. In this case, the electrons would transfer through the interfaces without being scattered and/or trapped at the grain boundaries, as in the case of single crystal STO. Moreover, in the absence of the grain boundary scattering, the carrier mobility in single crystalline STO is scattered by longitudinal optical phonon (*μ*_LO_), transverse optical phonon (*μ*_TO_), and ionized impurity scattering (*μ*_ii_)^37^. The total carrier mobility scattering can be determined by using Matthiessen’s rule Eq. (1) below.

1 $$\frac{1}{{\mu }_{total}}=\frac{1}{{\mu }_{LO}}+\frac{1}{{\mu }_{TO}}+\frac{1}{{\mu }_{ii}}$$

where

2 $${\mu }_{LO}=\frac{\hslash }{2\alpha \hslash {\omega }_{1}}\frac{e}{{m}_{P}}{(\frac{{m}_{e}^{\ast }}{{m}_{P}})}^{2}f(\alpha )({e}^{\hslash {\omega }_{1}/{k}_{B}T}-1)$$

3 $${\mu }_{TO}=\frac{\sqrt{2}\pi e{\hslash }^{3}\rho {\upsilon }_{s}^{2}}{{({m}_{e}^{\ast })}^{5/2}{\omega }_{TO}{D}_{lop}^{2}}\frac{({e}^{\hslash {\omega }_{TO}/{k}_{B}T}-1)}{\sqrt{E+\hslash {\omega }_{TO}}}$$

4 $${\mu }_{ii}=\frac{3{({\varepsilon }_{r}{\varepsilon }_{0})}^{2}{h}^{3}}{{Z}_{2}{m}^{\ast 2}{e}^{3}}\frac{n}{{N}_{i}}\frac{1}{{F}_{ii}({\xi }_{d})}$$

5 $${\xi }_{d}={(3{\pi }^{2})}^{1/3}\frac{{\varepsilon }_{r}{\varepsilon }_{0}{h}^{2}{n}^{1/3}}{{m}^{\ast }{e}^{2}}$$

6 $${F}_{ii}(\xi )=\,\mathrm{ln}(1+\xi )-\frac{\xi }{1+\xi }$$

where *ћ* = *h*/2*π* is the reduced Planck’s constant, *e* is the electron charge, *ћω*_1_ = 99 meV is the energy of the LO phonon mode involved in the scattering, *ћω*_*TO*_ = 5.95 meV is the energy of the TO phonon, *D*_lop_ = 9 eV is the optical phonon deformation potential, and *E* = *F*_E_ is the energy of electron, ${m}_{e}^{\ast }$ is the electron effective mass, *m*_e_ is the free electron mass, *m*_p_ = ${m}_{e}^{\ast }(1+{\rm{\alpha }}/6)$ is the polaron mass, *α* = 2.6 is the electron-phonon coupling constant, *f*(α) = 1 is a varying function, *k*_B_ is the Boltzmann constant, *ε*_0_ is the permittivity of vacuum, *ε*_r_ is the relative permittivity, *Z* = 2 is the charge of the ionized impurities, *n* is the carrier concentration, *F*_ii_(ξ) is a screening function, and *T* is the absolute temperature^38^. It is clearly evident that optimum RGO (RGO ≤ 0.7 wt%) incorporated samples exhibit single crystal-like mobility which is more than three times of magnitude than undoped STO sample. However, for higher RGO contents (RGO > 1 wt%) the mobility decreases, which is due to the agglomeration of the RGO at the grain boundaries, as shown in Figs 3 and 4. It implies that 0.7 wt% RGO content in undoped STO formed a well-distributed state and effectively suppressed the Sr sublimation and thereby reduced the double Schottky barrier height, and for the high RGO > 1 wt% the agglomerated RGO played as scattering centers, dominating the increase of mobility by reducing the double Schottky barrier height, which results in low carrier mobility. The calculated mobilities also suggest that longitudinal optical phonon (*μ*_LO_) is the dominant scattering mechanism in the homogeneously dispersed RGO ≤ 1 wt% composites, which is in excellent agreement with the evaluation of mobility measurements for the reduced single crystal STO^39^.

**Figure 6:**
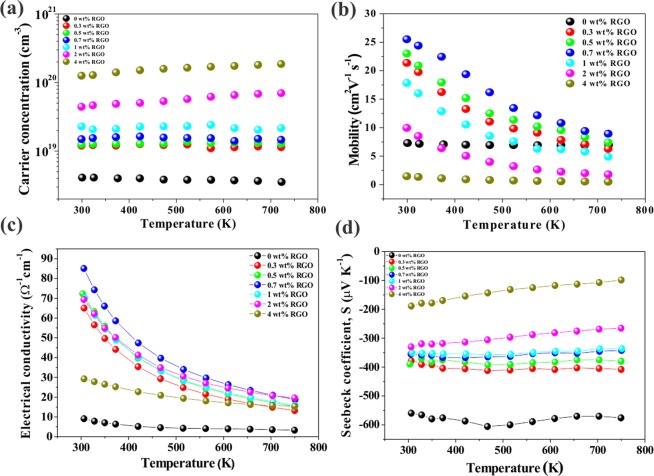
Temperature dependent (**a**) carrier concentrations, (**b**) mobilities, (**c**) electrical conductivities, and (**d**) Seebeck coefficients of the STO-RGO composites.

To investigate this excellent electronic transport properties of STO-RGO composite at high temperatures, temperature-dependent Hall measurements were performed as shown in Fig. 6(a,b). It is noteworthy to see that the RGO incorporation increases the carrier concentration in the full temperature range as presented in Fig. 6(a). The carrier concentrations in the homogeneously dispersed (up to 0.7 wt%) RGO samples were not depend on the temperature, indicating that these samples were degenerately doped. However, for agglomerated RGO contents (>1 wt%) a slight increase in the carrier concentrations is observed with increasing temperature. The origin of increase in the carrier concentration for homogeneously dispersed RGO samples is attributed to the release of the trapped electrons by reduction of the double Schottky barrier height and/or band alignment between RGO and STO. The electron carriers are originated from the oxygen vacancies, which are generated by SPS atmosphere (*p*O_2_~10^−5^ atm) and oxygen sublimation, which is enhanced by the RGO incorporation, at high temperatures. In the case of more than 1 wt% RGO, it is anticipated that the agglomerated RGO can make more reducing atmosphere during the sintering process, resulting in more oxygen vacancies in STO and thereof electrons for charge compensation, which is the origin of the further enhancement in carrier concentrations. This later situation is very similar to the recent reports in STO-RGO and Cu_2_O-RGO-In_2_O_3_ case^40,41^. In addition, the temperature dependent carrier concentration for high RGO contents shows a slightly different temperature dependent behavior, suggesting presumably additional thermal activation at the agglomerated RGO areas, i.e., the presence of the oxygen vacancies. Next, the temperature-dependent carrier mobilities of STO-RGO composites are presented in Fig. 6(b). It is interesting to see that the sample without RGO and RGO incorporated samples show different temperature-dependent behavior. The STO without RGO at high temperatures exhibited positive temperature coefficients of mobility, indicating the thermally activated process is dominant to overcome the double Schottky barrier at the grain boundary. However, as the RGO incorporated the samples show negative temperature coefficients of mobility in the whole temperature range, indicating that the grain boundary did not hinder the electron transport in the STO-RGO composites. In addition to this, as discussed above that with high RGO (>1 wt%) the agglomerated RGO act as a scattering center for the carrier mobility and consequently low carrier mobility.

Figure 6(c) represents the temperature dependent electrical conductivities (σ) of STO-RGO composites (0–4 wt% RGO). The electrical conductivity decreases with increase in temperature, exhibiting metallic behavior, i.e., negative temperature dependence due to the electron-phonon scattering, reduction of mobility. Furthermore, the electrical conductivity increases with increasing RGO content up to 0.7 wt% and then decreases with further increases in the RGO content. The value of electrical conductivity for the STO-0.7 wt% RGO composite at room temperature is about 9 times higher than that of the sample without RGO content. This enhanced electrical conductivity is attributed to simultaneous increases both in the carrier concentration and mobility. However, further increase in RGO content beyond the optimum concentration led to a reduction in the electrical conductivity, which is due to decrease in the carrier mobility by the agglomeration of RGO at the grain boundaries as discussed above.

The temperature dependent Seebeck coefficients (*S*) for STO-RGO composites (0–4 wt% RGO) are presented in Fig. 6(d). It can be seen that the Seebeck coefficients for all samples across the whole temperature range are negative, indicating the dominant charge carriers in the samples are electrons. Moreover, the Seebeck coefficient of pristine STO is slightly increased up to 470 K and then decreased linearly with increasing temperature. In the case of STO-RGO composites, the Seebeck coefficient is independent on temperature for RGO contents up to 1 wt% and then decreases linearly with increase in temperature for higher RGO contents. In addition to this, at room temperature, the decrease in the Seebeck coefficient with an increase in RGO contents may either come from an increase in the carrier concentration or decrease in the density of state (DOS) effective mass. To understand the dominant factor in the variation of Seebeck coefficient, the room temperature DOS effective masses of the samples were estimated using Pisarenko relation^26,42^ Eq. (7), as presented in S1(Supporting Information).

7 $$S=\frac{8\pi {k}_{B}^{2}}{3e{h}^{2}}{(\frac{\pi }{3n})}^{\frac{2}{3}}{m}_{d}^{\ast }T$$

where *k*_B_ is the Boltzmann constant, *h* is the Planck constant, *e* is the electronic charge, *n* is the carrier concentration, and *m*_d_* is the density of state (DOS) effective mass. It can be seen that the RGO incorporation increases both the DOS effective mass and carrier concentration as discussed above (see Fig. 6(a)). One should note that even the DOS effective mass increases, but the variation in the Seebeck coefficient are in contradictory to the DOS effective mass variation. This indicates that the Seebeck coefficient is governed by carrier concentration rather than DOS in STO-RGO composites.

**Figure 7:**
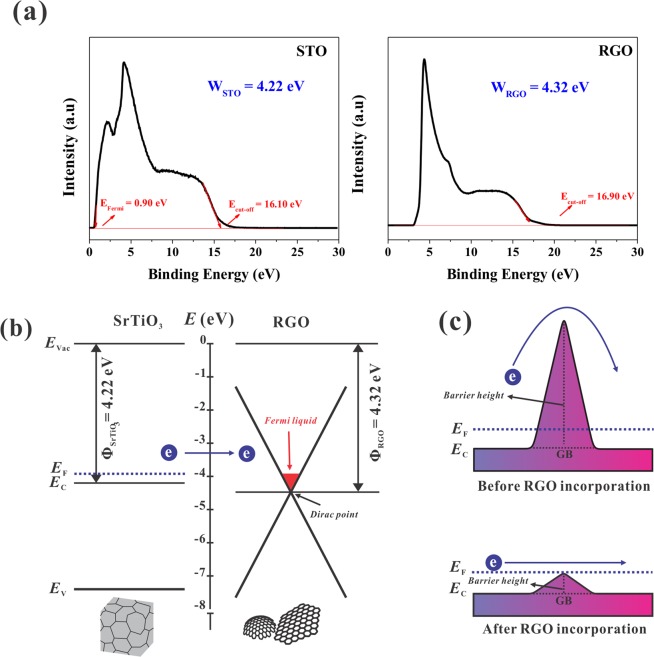
(**a**) UPS spectra of pristine STO and RGO (**b**) schematic of band alignment, and (**c**) schematic of double Schottky barrier height of the STO-RGO composites.

The mechanism of this excellent charge transport properties in STO-RGO composite can be elucidated by two possible models. The first is the concept of band alignments, to support this concept, the work function (Ф) of RGO and STO were measured using UPS, as shown in Fig. 7(a). In this study, the UPS spectra were measured with He-radiation (hν = 21.218 eV, where hν is the energy of the photons). The work function is determined by the equation: Ф = hν − (E_cut-off_ − E_Fermi_), where E_Fermi_ and E_cut-off_ are the Fermi level and binding energies of the secondary electron, respectively^43,44^. Here, the Fermi edges of RGO was set to zero^45^. The work function of STO and the RGO is 4.22 eV and 4.33 eV, respectively, which is similar to the reported work^46,47^. In this case, STO-RGO interfaces are formed, as shown in the schematic Fig. 7(b), where the Fermi level in the RGO rises to the Fermi level of STO and the electrons moved from STO to RGO without any grain boundary scattering like single crystal as shown in Fig. 5. In addition to this, this behavior also increases the carrier concentration in STO-RGO composites. This kind of behavior is similar to other oxide-graphene composites^48–51^.

**Figure 8:**
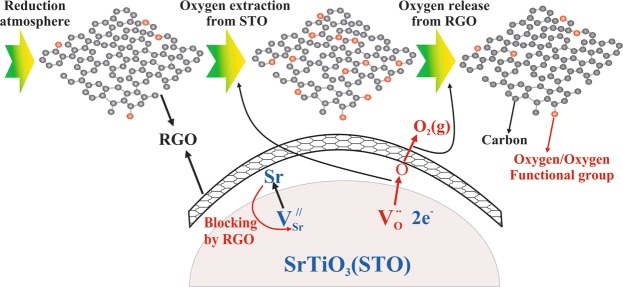
Schematic diagram of RGO coated STO grains, blocking Sr sublimation and pumping out oxygen.

The second concept is the suppression of double Schottky barrier height by RGO incorporation at the grain boundaries as shown in the schematic Fig. 7(c). Mizoguchi *et al*. have performed Electron Energy Loss Near-edge Structures (ELNES) together with the first-principles calculations to identify native defects at the vicinity of STO grain boundary^52^. They found that Sr-vacancies are concentrated in the vicinity of the grain boundary. Moreover, Kim *et al*. also used a combination of EELS spectra and first-principles calculations, and they observed that vacancies at the grain boundaries provide an explanation of the microscopic origin of the double Schottky barriers which dictate the electrical behavior of polycrystalline oxides^53^. Based on the aforementioned observations, it is expected that by RGO incorporations, covers the STO grains, inhibit the Sr-vacancies generation, and as a result, the double Schottky barrier height is reduced. This reduction helps the electrical behavior of STO-RGO composite. As aforementioned, the electron carriers are originated from the oxygen vacancies, which are generated by SPS atmosphere (*p*O_2_~10^−5^ atm) and oxygen sublimation at high temperatures. It is believed that RGOs at the grain boundaries prevent the Sr sublimation, but enhance the oxygen sublimation at the reducing atmosphere, as shown in schematic Fig. 8.

**Figure 9:**
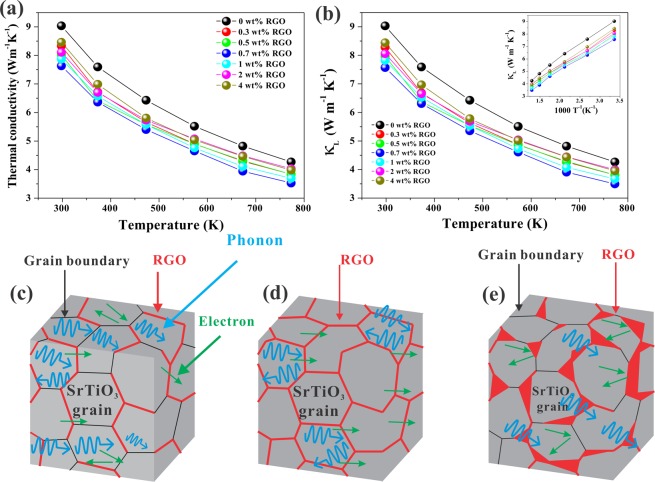
Temperature dependent (**a)** total thermal conductivity, and (**b**) lattice thermal conductivity of the STO-RGO composites. The inset in (**b**) shows the plot of *κ*_L_ vs. *T*^−1^ of the STO-RGO composites and (**c**–**e**) is the schematic representation of various RGO content in STO matrix.

Figure 9(a) displays the temperature dependence of the total thermal conductivity (*κ*) of STO-RGO composites (0–4 wt% RGO). The thermal conductivities of STO-RGO composites are lower than that of pristine samples. It is noted that with a low content of RGO, the thermal conductivity rapidly decreases and reaches lowest values for 0.7 wt% RGO sample. However, with further increase in RGO content the thermal conductivity increases, but remains below than that of the pristine samples in the entire temperature range. Figure 9(b) shows the lattice thermal conductivity, which can be obtained by using Wiedemann-Franz relationship^54^. It can be seen that the central involvement to the total thermal conductivity comes from the lattice thermal conductivity. The lattice thermal conductivity was plotted as a function of *T*^−1^, as shown in the inset in Fig. 9(b), and shows a linear relationship with *T*^*−*1^. This linear correlation indicates that the lattice thermal conductivity is affected mainly by Umklapp scattering^55^. In addition to this, it is interesting to see that with increasing RGO content from 0.3 to 0.7 wt%, i.e., the well-dispersed RGO samples, the lattice thermal conductivity shows a reduction tendency, which suggests that interface phonon scattering effect by RGO at the grain boundaries is dominant. However, further increase in the RGO content (RGO > 0.7 wt%), i.e., agglomerated samples, shows an increase in the lattice thermal conductivity. The reason may be attributed to the ultra-high thermal conductivity of agglomerated RGO and/or uncovered RGO grain boundaries which may provide an effective way for phonon transfer, and as a result overall thermal conductivity is increased. Nevertheless, it is still lower than that of the pristine sample in the whole temperature window. This behavior is very analogous to the previous reports^15,56^.

Figure 9(c–e) schematically summarize the distribution of RGO in STO matrix and its effect on the thermoelectric properties. As discussed above that RGO is used to cover the grain boundary to weaken the double Schottky barrier and release the trapped electrons from the grain boundaries. Figure 9(c) illustrates schematically that for low RGO contents, i.e., below 0.7 wt%, the RGO content is not enough to cover all grain boundaries of the STO matrix and as a result, some of the uncovered grain boundaries could be the scattering center for carrier mobility. However, in case of 0.7 wt% RGO sample, as illustrated schematically in Fig. 9(d), (optimum concentration), most of the grain boundaries in STO matrix were covered with RGO and thereby the role of the double Schottky barrier is negligible like single crystal STO. The STO-RGO interface also acts as a phonon scattering center for thermal conductivity and as a result lower lattice thermal conductivity. In contrast, for high RGO contents, i.e., RGO > 0.7 wt%, the RGOs are agglomerated at the grain boundaries in the STO matrix, and some of the grain boundaries have missed by the RGO as shown in the schematic Fig. 9(e). These agglomerated RGO at the grain boundaries and/or uncovered RGO grain boundaries act as scattering centers for carrier mobility, which results in low carrier mobility. However, the agglomerated RGO at the interfaces and/or uncovered RGO grain boundaries allow phonons to move across the interfaces and/or grain boundaries, which results in high thermal conductivity as compared to other STO-RGO composite samples with lower RGO contents.

**Figure 10:**
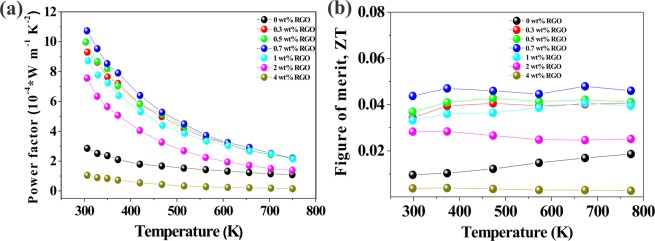
Temperature dependent (**a**) powder factors and (**b**) ZT values of the STO-RGO composites.

The power factor (PF = σS^2^) is presented as a function of temperature in Fig. 10(a). The STO-0.7 wt% RGO composite exhibits the highest power factor about more than three times of magnitude than pristine STO, which is mainly ascribed to the high electrical conductivity and Seebeck coefficient. The gained PF in this work is equivalent to the PF obtained high donor-substitution in STO^35^. Temperature dependencies of dimensionless figure of merit (ZT) for all STO-RGO composites (0–4 wt% RGO) are shown in Fig. 10(b). A significantly enhanced power factor in the optimum composition (0.7 wt% RGO), coupled with the lowest thermal conductivity, gives rise to about five times enhanced ZT as compared to pristine STO. The increase in the ZT demonstrates that carefully controlled grain boundary of oxide candidate materials can enhance the ZT up to an order of magnitude combining with metal ion-site substitutions, which lower further the thermal conductivity, in oxides.

## Conclusion

In conclusion, the effects of double Schottky barrier at grain boundary on the transport properties of undoped STO were studied and found that the double Schottky barrier impedes the transport properties in undoped STO. Our results demonstrated that by incorporating optimum RGO content, lowering the double Schottky barrier, simultaneously increases the carrier concentration and mobility which exhibits single crystal-like mobility in undoped STO. In addition to this, at the same time STO-RGO interface acts as a phonon-scattering center, which results in low lattice thermal conductivity. The strategies used in this work are broadly important and have the possibility for increasing the TE properties of candidate oxide TE materials.

## Materials and Methods

### Synthesis of STO/RGO composite

In this experiments STO particles were synthesized by conventional solid-state reaction method, using TiO_2_ (Sigma Aldrich, 99.8%) and SrCO_3_ (Sigma Aldrich, ≥99.9%) as starting materials. These powders were mixed according to the stoichiometric ratio. Next, the powder were ball-milled for 24 h in ethanol. The dried slurries were calcined at 1373 K for 3 h and then ball-milled again to enhance homogenization. After complete drying, micronsized STO powders (5 g) and the GO (0–4 wt%) were mixed with 200 ml of N,N-dimethylformamide (DMF, C_3_H_7_NO, Sigma Aldrich) in a flask yielding STO powders coated with the GO, and an ultrasonic treatment was performed for 10 min to disperse the STO powders and GO effectively. The hydrazine monohydrate (0.2, 0.6, 1, 1.4, 2, 4, and 8 ml for 0.1, 0.3, 0.5, 0.7, 1, 2, and 4 wt% GO, respectively) as a reducing agent for the GO was dropped in the flask, and the mixture was kept at 80 °C for 1 h. After the chemical reaction, the mixture aged at room temperature for 24 h was washed with DMF, and dried in a rotary oven at 80 °C. Finally, the obtained STO-RGO composite powders were loaded into a graphite mold of 10 mm diameter and consolidated into a bulk composite by spark plasma sintering (SPS). The mold was heated up to 1573 K under 60 MPa of pressure for 15 min in a 10^−3^ torr vacuum with a heating rate of 100 °C/min.

### Characterization

X-ray diffraction analysis of the bulk composite samples was analyzed using Rigaku D/MAX-2500/PC with Cu K*α* radiation. The microstructure of the composites was examined by using a high-resolution transmission electron microscope (HRTEM, JEM- 4010, JEOL) and scanning electron microscopy (SEM, Verios 460 L, FEI). Rectangular sample (2 × 2 × 9 mm^3^) were cut for the estimation of electrical conductivity and Seebeck coefficient, which were measured by using a four-point probe (TPMS, ZEM-3). High temperature charge transport performance were measured up to 730 K by using a high-temperature Hall measurement (HT-Hall, Toyo Corporation, ResiTest 8400). A circular disc specimen of 1 mm in thickness and 10 mm in diameter was used for thermal diffusivity measurement using a laser flash method (DLF-1300, TA instrument). The density was measured by Archimedes-principle. Thermal conductivity (κ) was calculated from the relation, diffusivity (*α*) × density (*ρ*) × specific heat capacity (*C*_*p*_).

## Supplementary information

Supporting information
