# Supplementary material for: Grain Boundary Interfaces Controlled by Reduced Graphene Oxide in Nonstoichiometric SrTiO3-δ Thermoelectrics
Source: Sci Rep. 2019 Jun 13;9:8624. doi: 10.1038/s41598-019-45162-7 (PMC6565681; doi:10.1038/s41598-019-45162-7)
Supplement: Supplementary file 1 — Supporting information [file 41598_2019_45162_MOESM1_ESM.docx]

**Grain Boundary Interfaces Controlled by Reduced Graphene Oxide in Nonstoichiometric SrTiO_3-_*_δ_* Thermoelectrics**

Jamil Ur Rahman,^a,b^ Nguyen Van Du,^a,b^ Woo Hyun Nam,^a^ Weon Ho Shin,^a^ Kyu Hyoung Lee,^c^ Won-Seon Seo,^a^ Myong Ho Kim,^b^ and Soonil Lee^b,^^[[1]](#footnote-1)^

^a^ Energy & Environmental Materials Division, Korea Institute of Ceramic Engineering & Technology, Jinju 52861, South Korea

^b^ School of Materials Science and Engineering, Changwon National University, Changwon 51140, South Korea

^c^ Department of Materials Science and Engineering, Yonsei University, Seoul 03722, South Korea





**S1**. Seebeck coefficients as a function of carrier concentration. The dashed lines represent the theoretical Seebeck coefficients calculated using a degenerate semiconducting model.

1. Corresponding author: leesoonil@chagwon.ac.kr

   Tel: +82-55-213-3717, Fax: +82-55-262-6486 [↑](#footnote-ref-1)
